# Sucrose‐induced metabolic syndrome differentially affects energy metabolism and fiber phenotype of EDL and soleus muscles during exercise in the rat

**DOI:** 10.14814/phy2.16126

**Published:** 2024-07-12

**Authors:** Rodríguez‐Correa Eduardo, Carvajal Karla

**Affiliations:** ^1^ Laboratorio de Nutrición Experimental Instituto Nacional de Pediatría Ciudad de México Mexico

**Keywords:** exercise, metabolic syndrome, metabolism, myosin, skeletal muscle

## Abstract

Molecular mechanisms associated to improvement of metabolic syndrome (MetS) during exercise are not fully elucidated. MetS was induced in 250 g male Wistar rats by 30% sucrose in drinking water. Control rats receiving tap water were controls, both groups received solid standard diet. After 14 weeks, an endurance exercised group, and a sedentary were formed for 8 weeks. The soleus and extensor digitorum longus (EDL) muscles were dissected to determine contractile performance, expression of myosin heavy chain isoforms, *PGC1α*, *AMPKα2*, *NFATC1*, *MEF2a*, *SIX1*, *EYA1*, *FOXO1*, key metabolic enzymes activities. Exercise mildly improved MetS features. MetS didn't alter the contractile performance of the muscles. Exercise didn't altered expression of *PGC1α*, *NFATC1*, *SIX1* and *EYA1* on MetS EDL whereas *NFATC1* increased in soleus. Only citrate synthase was affected by MetS on the EDL and this was partially reverted by exercise. Soleus *α‐*ketoglutarate dehydrogenase activity was increased by exercise but MetS rendered the muscle resistant to this effect. MetS affects mostly the EDL muscle, and endurance exercise only partially reverts this. Soleus muscle seems more resilient to MetS. We highlight the importance of studying both muscles during MetS, and their metabolic remodeling on the development and treatment of MetS by exercise.

## 1 INTRODUCTION

Metabolic syndrome (MetS) is one of the main current health problems worldwide as it has been shown to affect around a billion people (Saklayen, 2018). Several international organizations have defined MetS as the coincident presence of three or more different pathological conditions like abdominal obesity, glycemia alterations, arterial hypertension and hyperlipidemia, among others. These conditions are considered risk factors to develop type 2 diabetes mellitus (T2DM), cardiovascular diseases and some types of cancer (Russo et al., 2008; Sattar et al., 2008). The World Health Organization recommends increase of physical activity levels and healthier modifications on the diet, in order to prevent and treat MetS (World Health Organization 2022).

Several rodent models are used to develop and study the MetS. In this sense, it has been shown that the hypercaloric high‐sucrose model induces insulin resistance (IR) (determined by HOMA index, glucose and insulin tolerances, glucose uptake by peripheral tissues and fasting insulin levels), hyperlipidemia, adiposity, obesity and impaired lipidic and glycolytic metabolisms (Rodríguez‐Correa et al., 2020).

The skeletal muscle (SM) plays an important role in the metabolic regulation of the organism, as it represents around 40% of the total mass. Besides, it accounts for 22% of the basal metabolic rate of whole body (McClave & Snider, 2001). Also, it has a relevant role on glucose clearance after eating (Nuutila et al., 1995), and during exercise, it improves the carbohydrate and lipid metabolisms by reverting most of the conditions related to MetS (Fujii et al., 2000; Yan et al., 2011).

AMPK and PGC1*α* have shown impaired activity or diminished expression during MetS (Feige & Auwerx, 2007; Mootha et al., 2003; Patti et al., 2003). The molecules that induce changes into faster phenotypes are less studied, with discrepancies among reports, probably because they have been evaluated under variable conditions. Nevertheless, it has been described that EYA1 recruits SIX1 to the nucleus and in turn, SIX1 promotes transcription of a long non‐coding RNA (lnc‐MyHC), which promotes the transcription of the 2b and 2x MyHC types; even though, its absence does not induce the expression of type 1 MyHC (Girgis, 2018; Gordon et al., 2012; Hetzler et al., 2014). FOXO1 plays an important role on SM embryonic development and differentiation (Xu et al., 2017), it also seems to play a role on differentiation from type 1 fibers into type 2 (Shi et al., 2014; Xu et al., 2017; Yuan et al., 2011), and may also been involved in the response of SM to endurance exercise, as it has been shown that its expression decreases during this condition (Yuan et al., 2011).

Different interactions between these factors may contribute to the alterations seen in MetS and may also be responsible for the benefits achieved during different types of exercise. Endurance exercise is known to enhance oxidative metabolism and induce the expression of slower MyHC (Lin et al., 2002; Little et al., 2010); therefore, it is thought that the molecular mechanisms responsible for this improvement are the same that mitigate the effect of MetS, usually implying an increase in oxidative metabolism and fiber type transition towards a slower phenotype in the slow muscles. For these reasons, the research has mainly been focused on the role of these slow and oxidative muscles towards the development of MetS; in general, endurance exercise is recommended to prevent and treat it.

Despite the available information, there are knowledge gaps about the interaction between factors that are responsible for the enhancement of energetic metabolism and the impact on mechanical properties of the muscles that have been impaired during MetS. Especially, the fiber type depends on several factors that vary among muscle types. Therefore, the aim of this study was to describe the mechanical properties of fast and slow SMs of rats with sugar‐induced MetS and the impact in their metabolic and molecular phenotype after an endurance exercise protocol.

## 2 BIOETHICAL AND BIOSAFETY ASPECTS

Animal's use observed the guidelines in accordance with Official Mexican Law (NOM‐062‐ZOO‐2002), institutional regulations and the NIH Guide for the Care and Use of Laboratory Animals. As well, the institutional ethics in animals (IACUC), research biosafety and research committees, registered at the Office for Human Research Protection of the NIH (http://ohrp.cit.nih.gov/search/search.aspx) with numbers IRB00013675 and IRB00013674, approved the present protocol under the number INP 021/2017.

## 3 MATERIALS AND METHODS

**FIGURE 1:**
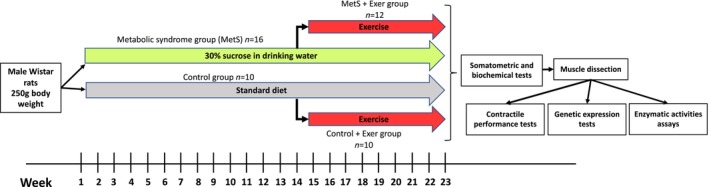
Schematic representation of the experimental strategy.

A schematical representation of the general methodology is represented in Figure 1.

### 3.1 Animals and diet

To induce MetS, 29 male Wistar rats weighing 250 g were subjected to a 24‐week period of hypercaloric diet where 30% sucrose was added to their drinking water; a control group (*n* = 26) received sugar‐free tap water. Chow diet was given ad libitum for both groups. Calories provided by macronutrients of the chow diet for all groups are as follows: Protein 26.6%, fat 16.5%, and carbohydrates 56.9% (Formulab Diet 5008, PMI, USA). All animals were housed in a controlled environment with 12 h light/dark cycles at 25°C. Three animals were housed per cage.

In order to determine the effects of endurance exercise on the MetS, at week 14, the animals from both groups were randomly assigned to endurance exercise training or remained sedentary.

### 3.2 Training protocol

To reduce the psychological effect of the treadmill, at week 14 of sucrose consumption, the individuals selected for training (control and sucrose drinking likewise) started an acclimation period on a treadmill (LE87OOC, Panlab, USA), consisting of 10 min running at increasing speed going up to 15 cm/s. At the end of this week, to evaluate initial aerobic and physical capacity, a maximal performance test was executed, running speed was increased by 5.0 cm/s every 3 min until exhaustion (the moment in which the animal refused to keep running by staying for more than 10 s on the electric grid), when VO_2 max_ was determined. From the next 8 weeks, animals were trained 5 day per week on the treadmill at a constant speed of 0.2 m/s, which corresponded to an intensity superior to 60% of their VO_2 max_ (data not shown) for 50 min, with the band inclined by 10°. At the end the training period, the performance test was repeated to determine the aerobic capacity. During every session the chamber gas composition was determined using a LE405 gas analyzer (Panlab, USA) in order to record the VO_2 max_ and respiratory quotient (RQ).

### 3.3 Glucose tolerance test

To determine IR, at week 24 animals were fastened for 6 h and then injected intraperitoneally with a 50% glucose solution (Pisa, México) at a dose of 1.0 g/kg body weight. This dose was selected because 2.0 g/kg induced very high values that the glucometer was not able to measure adequately, probably due high IR in our animals. The blood was extracted from the tail vein and the glucose concentration was determined using a portable glucometer (Accutrend Plus, Roche, Switzerland) every 15 min for the first hour and then every hour until minute 180.

### 3.4 Somatometric parameters

Three days after the last performance test all animals were fastened for 6 h and anesthetized with sodium pentobarbital at 30 mg/kg body weight (Pisabental, Pisa, México). To determine MetS parameters the animals were euthanized by heart removal during anesthesia and a drop of blood was extracted and analyzed to measure the triglyceride (TG) concentration by using a personal analyzer (Accutrend Plus, Roche, Switzerland). After that, retroperitoneal fat was dissected thoroughly and weighted on an analytical balance. Also, the tibia was separated and its length was used as reference of the animal's size.

Five control animals that had TG values higher than 250 mg/dL and fat percentage higher than 5% were excluded from further analysis; as well, one rat from MetS group with TG values lower than 250 mg/dL and fat percentage lower than 5% were excluded.

### 3.5 Muscle dissection

EDL and soleus muscles from the posterior legs were dissected and quickly preserved in buffered Krebs solution (NaCL 130 mM, KCl 5 mM, MgCl_2_ 2 mM, NaHCO_3_ 15 mM, Na_2_HPO_4_ 1 mM, glucose 11 mM and CaCl_2_ 0.33 mM pH = 7.4) constantly bubbled with a mixture of oxygen and carbon dioxide (5% and 95%, respectively) and maintained at 25°C. The muscles from one leg were used for the electrical stimulation protocol to evaluate mechanical properties, while those from the opposite leg were quickly frozen in liquid nitrogen and then stored at −70°C for further biochemical and molecular analyses to determine molecular and metabolical properties.

### 3.6 Electrical stimulation protocol

In order to determine maximum strength, fatigue resistance and force recovery after fatigue, isolated muscles were tied up by the tendons and placed into a glass isolated‐organ chamber filled with bubbled Krebs solution. One end of the muscle was fixed to an isometric force transducer (ADInstruments, USA) connected to a signal acquisition system (Power lab, ADInstruments, USA) and data were plotted and analyzed with Lab chart software (ADInstruments, USA). Two Platine electrodes were placed in contact with the muscle and an electrical stimulator (588 GRASS instruments, USA) was used to induce muscle contractions. Single twitch pulses of 0.6 ms at 75 Hz were used to determine the optimal voltage and length needed to obtain maximal tension (Ramirez‐Soto et al., 2019).

To determine maximal isometric force, a tetanic stimulation of 75 Hz for 3 s was given and after that, the muscle was allowed to rest for 10 min. To determine fatigue resistance, periodical tetanic stimulations of 75 Hz for 350 ms were given every second until the force reached 50% of the initial value. Finally, after 25 min at rest, another tetanic stimulation was given and compared to the force produced during the initial fatigue tetanus; this allowed us to determine the percentage of force recovered. During resting periods new Krebs solution was flushed into the chamber. After the stimulation protocol, muscles were dried out on a paper tissue and were weighted on an analytical balance to determine weight changes.

### 3.7 Gene expression assays

Genetic assays were selected to determine changes at the transcription level. For RNA extraction, 40 mg of frozen muscle was placed in Trizol (Invitrogen, Spain), homogenized with an electrical homogenizer (ProScientific, Oxford) and let rest on ice. After that, chloroform (Merck, Germany) was added and after centrifugation at 8000 rpm for 45 min, the supernatant was recovered. After that, 70% ethanol was added and centrifuged for 30 min at 7500 rpm and 4°C. The pellet was washed with 70% ethanol three times and resuspended in sterile RNAses free water (AMRESCO, USA). The RNA concentration and purity were determined by a spectrophotometric analysis (NanoDrop, USA), only samples with 260/280 nm and 260/230 nm coefficients higher than 1.9 were used. To obtain cDNA, 200 ng of RNA were retrotranscribed following the kit manufacturer's instructions (Invitrogen, USA) in a thermocycler (Veriti, Applied Biosystems, USA). Concentration and purity were determined by wavelength analysis (NanoDrop, USA), samples with 260/280 nm and 260/230 nm coefficients higher than 1.8 were used.

For the expression assays, primers coding for *MyHC1*, *MyHC2a*, *MyHC2x*, *MyHC2b*, *PGC1α*, *FOXO1*, *MEF2a*, *NFTATc1*, *Six1* and *Eya1* were added to a mixture of SYBR GREEN master mix with (Thermo Fisher scientific, USA) and 250 ng of cDNA per sample. The annealing and amplification temperatures, primer sequences and respective references are described in Table 1 (Azad et al., 2016; Calabria et al., 2009; Colom et al., 2007; Gordon et al., 2012; Guan et al., 2016; Raciti et al., 2010).

**TABLE 1:** Primers used for the PCR assays.

Gene	Forward sequence	Reverse sequence	Reference	Annealing temperature (°C)	Annealing duration (s)
**MyHC_1**	ggcctgaatgaagagtagat	gtgtttctgcctaaggtgct	Azad et al. (2016)	55	30
**MyHC_2a**	atgacaactcctctcgctttgg	ttaagctggaaagtgacccgg	Azad et al. (2016)	58	30
**MyHC_2x**	ccaatgagactaagacgcctgg	gctatcgatgaattgtccctcg	Azad et al. (2016)	58	60
**MyHC_2b**	gaacacgaagcgtgtcatcca	aggtttcgatatctgcggagg	Azad et al. (2016)	58	60
**PGC1α**	atgagaagcgggagtctgaa	gcggtctctctcagttctgtcc	Guan et al. (2016)	60	60
**MEF2a**	cctggaatgctgtctctgg	Tggctctgtcttaatgttgatg	Raciti et al. (2010)	57.5	60
**NFATC1**	tgaacctctcacgctacag	caccacccagtataccagc	Calabria et al. (2009)	55.9	60
**FOXO1**	tcctcgaaccagctcaaacg	ggcggtgcaaatgaatagcaag	Azad et al. (2016)	58	30
**SIX1**	cgaggccaaggaaagggag	actcctcttctgagctggacatg	Gordon et al. (2012)	61	60
**EYA1**	gtacagctaccagatgcgtagcag	atgtagtgtgctggatacggcgag	Gordon et al. (2012)	62	60
**18S**	gaggtgaaattcttggaccgg	cgaacctccgactttcgttct	Colom et al. (2007)	72	30

The qPCR reactions were run in a thermocycler (StepOne, Applied Biosystems, USA) and Ct levels were analyzed using the software provided with the equipment. For the expression of *AMPK* alpha 2 subunit, pre designed Taqman genes were used (Hs00178903 for *PRKAA2* and 4333760F for *18S*, Applied Biosystems) and manufacturer's instructions were followed thoroughly. the reactions were run in a thermocycler (7500, Applied Biosystems, USA). Ribosomal *18S* rRNA was used as an endogenous control for the target genes and the relative expression of each gen was normalized against it. Mean values from control muscles (standard diet and not exercise) were used as reference for the experimental conditions to assess the fold change induced by MetS or exercise for each gene using the 2^−ΔΔCt^ method as described by Livak (2001). As we found high dispersion in the control groups, we decided to add them into the graphs in order to help readers to take it into account when interpreting the data here presented.

### 3.8 Enzymatic activity assays

To determine the metabolic properties of the muscles the activities of key glycolytic and oxidative enzymes were determined. Around 60 mg of frozen tissue was homogenized in cold lysis buffer that contained HEPES 50 mM pH 7.5, KCl 50 mM, EDTA 1 mM, EGTA 1 mM, β‐glycerolphosphate 5 mM, Tritón X‐100 0.1%, NaF 50 mM and NaPPi 5 mM. The protein concentration was determined by the Lowry method. For activity assays, reaction was started by addition of the protein homogenate.

Enzymatic activities protocols were performed as previously described (Carvajal et al., 2003, 2005) The activity of the hexokinase‐2 (HK‐2) was assayed in a medium that contained HEPES 20 mM pH 7.5, MgCl_2_ 15 mM, glucose 2 mM, glucose‐6 phosphate dehydrogenase (1 IU), NADP 1 mM and ATP 10 mM. The activity was spectrophotometrically followed by NADPH formation at 340 nm. The activity of the α‐ketoglutarate dehydrogenase (αKGDH) was assayed in medium that contained HEPES 40 mM pH 7.1, MgCl_2_ 1 mM, TPP 0.2 mM, NAD 2 mM, coenzyme A 1 mM, ditiotreitol 1 mM, rotenone 1 μM, and α‐ketoglutarate 10 mM. The activity was spectrophotometrically followed by NADH formation at 340 nm. Phosphofructokinase 1 (PFK‐1) activity was assayed in a medium that contained HEPES 20 mM pH 7.5, KCl 150 mM, EDTA 1 mM, MgCl_2_ 15 mM, NADH 1 mM, fructose‐6‐phosphate 2 mM, ATP 5 mM, aldolase, triosephosphate isomerase (0.5 IU) and glycerol‐3‐phosphate dehydrogenase (2 IU). The activity was spectrophotometrically followed by NADH consumption at 340 nm. Citrate synthase was assayed in a medium that contained Tris 100 mM, acetyl‐coenzyme A 0.5 mM, oxaloacetate 0.5 mM and DTNB 0.1 mM and was spectrophotometrically followed by TNB formation at 412 nm. Finally, the activity of the pyruvate kinase (PK) was assayed in a medium that contained HEPES 20 mM pH 7.5, KCl 150 mM, EDTA 1 mM, MgCl_2_ 2.5 mM, ADP 1 mM, phosphoenol‐pyruvate 1 mM, NADH 0.5 mM, and lactate dehydrogenase (2 IU).

### 3.9 Statistical analysis

Following previous studies (Farsijani et al., 2021; Szaroszyk et al., 2022) all data were analyzed by the Kruskal–Wallis test (KW) to determine statistical differences among groups. After that, Mann–Whitney test (MW) was used to compare medians between individual groups. For both tests, a Bonferroni correction was applied, and significance was set at *p* < 0.01. Values that exceeded at least 10 times the highest value for that group were excluded from the statistical analysis, as they were considered outliers, even though they were presented as crossed dots on the graphs. GraphPad prism® V5.01 was used for analysis and graphs.

## 4 RESULTS

### 4.1 Somatometric and in vivo biochemical parameters

Table 2 shows the results of the biochemical and somatometric parameters of the animals at week 23. All parameters showed statistically significant differences among groups when using the KW test. The changes of these parameters indicate that the experimental groups developed MetS, since they presented a significant increase of the retroperitoneal fat deposits and fasting TGs, as already reported (Camacho‐Castillo et al., 2021). Even when statistical difference was not found on the AUC of the glucose tolerance test and on the glucose levels after 3 h, due the sucrose water consumption, it has already been reported on this model that animals suffer from IR (Camacho‐Castillo et al., 2021). A trend for increased weight gain was also observed, although it did not reach statistical significance. No changes were induced by the MetS on the muscles mass.

**TABLE 2:** Biochemical and somatometric parameters at week 23.

Measurement	Control	MetS	MetS + exercise	Control + exercise
Retroperitoneal fat % (g/cm) ($)	2.9 ± 0.2 (*n* = 10)	5.2 ± 0.5*** (*n* = 16)	3.4 ± 0.4^††^ (*n* = 10)	2.6 ± 0.3 ^†††^ (*n* = 10)
Triglycerides (mg/dL)	193 ± 22 (*n* = 10)	377.4 ± 32*** (*n* = 16)	282.9 ± 37 (*n* = 10)	177 ± 24 ^†††^ (*n* = 8)
Plasma glucose levels after 3 h of glucose tolerance test (mg/dL)	118 ± 14 (*n* = 10)	163 ± 16 (*n* = 13)	129 ± 12 (*n* = 12)	94 ± 6 ^‡‡^ (*n* = 10)
Area under curve from glucose tolerance test (mg·h/dL)	36,450 ± 4119 (*n* = 10)	49,768 ± 6265 (*n* = 13)	49,322 ± 3065 (*n* = 10)	29,771 ± 2412^‡‡‡^ (*n* = 10)
Weight gain from week 16 to 23 (g)	63 ± 11 (*n* = 10)	100 ± 24 (*n* = 15)	25 ± 7 ^†††^ (*n* = 12)	−7 ± 9^*** †††^ (*n* = 10)
Fasting blood glucose (mg/dL)	82 ± 7 (*n* = 10)	84 ± 4 (*n* = 15)	102 ± 5 (*n* = 12)	78 ± 5 ^‡‡^ (*n* = 10)
EDL weight (mg/cm) ($)	6 ± 0.2 (*n* = 10)	5.6 ± 0.2 (*n* = 9)	5.4 ± 0.2 (*n* = 10)	6.3 ± 0.1 ^‡‡^ (*n* = 10)
Soleus weight (mg/cm) ($)	6.4 ± 0.3 (*n* = 10)	6.5 ± 0.3 (*n* = 12)	5.8 ± 0.3 (*n* = 10)	7.3 ± 0.3 ^‡‡^ (*n* = 10)

*Note*: KW test showed statistically significant difference among groups for all parameters (*p* < 0.01). Paired comparisons were made with MW test. ***p < 0.001 versus Control. ^††^ *p* < 0.01 and ^†††^ *p* < 0.001 versus MetS. ^‡‡^ *p* < 0.01 and ^‡‡‡^ *p* < 0.001 MetS+ Excersice. $ = normalized against the length of the tibia. Values are means ± SEM.

Endurance exercise fully reverted the effect of the MetS on the retroperitoneal fat, but was not able to improve the glucose clearance during the glucose tolerance test (AUC), suggesting mild improvements induced by the endurance exercise on the IR. Endurance exercise significantly reduced the weight gain of the MetS animals (Rodríguez‐Correa et al., 2020; Sousa et al., 2018) (Table 2).

Control exercised animals did not significantly changed adiposity, TG, glucose clearance, fasting glucose levels or muscle mass. Soleus muscle showed a trend for an increased mass and these animals showed significant lower body weight gain (Table 2).

### 4.2 Effect of endurance training on metabolic and physical performance in MetS animals

**FIGURE 2:**
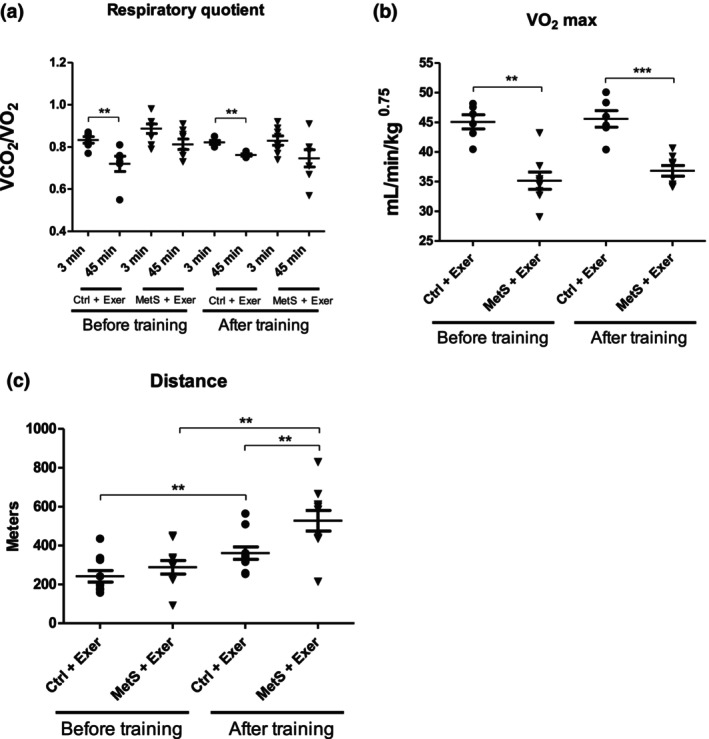
Physical and metabolic features, before and after 8 weeks of endurance training. Respiratory quotient during first and last training sessions at the 3rd and 45th minute (a), VO_2 max_ (b), and maximum distance ran during the performance test (c). KW test showed statistically significant difference among groups for all parameters (*p* < 0.01). Paired comparisons were made with MW test. Medians represented by the bar. *n* = 6–10. **p < 0. 01, ***p < 0.001.

Figure 2 shows the physical and metabolic adaptations induced by 8 weeks of endurance training. MetS animals showed no changes in RQ at the first training session when compared to the controls (Figure 2a), implying that MetS had no effect on this metabolic parameter.

As for the aerobic capacity, MetS animals showed reduced VO_2 max_ when compared to the controls (Figure 2b) even when they were able to run the same maximum distance until exhaustion (Figure 2c), however, aerobic capacity was not improved after 2 months of training (Figure 2b), this suggests that this exercise intensity may not be enough to achieve an increased aerobic capacity.

Interestingly, MetS animals were able to run a longer distance than the control + exercise group during the performance test at the end of training, although the initial run capacity was unaffected (Figure 2c).

### 4.3 Skeletal muscle mechanical properties

Table 3 shows the mechanical properties of muscles. Given the astringency used in the statistical tests, we found no significant differences on the evaluated mechanical properties of the muscles. However, MetS showed a trend for an increased maximal force produced in EDL muscles and increased force recovery of both muscles. Endurance exercise was not able to fully revert the observed trends. EDL muscles of the MetS + Exercise animals also showed a trend for increased fatigue resistance capacity and an increased force recovery when compared with the sedentary MetS muscles. Also, in MetS animals exercise showed a trend for increased fatigue resistance of soleus muscle when compared to the control. As for the MetS + Exercise animals, soleus muscle force recovery seems to be partially reverted (Table 3).

**TABLE 3:** Mechanical properties of EDL and soleus muscles.

Muscle	Control	MetS	MetS + exercise	Control + exercise
Maximum tetanic stress (N/g)				
EDL	0.87 ± 0.1 (*n* = 10)	1.16 ± 0.08 (*n* = 11)	1 ± 0.08 (*n* = 12)	1 ± 0.1 (*n* = 8)
Soleus	0.69 ± 0.1 (*n* = 10)	0.9 ± 0.09 (*n* = 11)	0.9 ± 1 (*n* = 12)	0.7 ± 0.09 (*n* = 10)
Fatigue resistance (s)				
EDL	96 ± 7 (*n* = 8)	108 ± 8 (*n* = 9)	137 ± 9 (*n* = 11)	107 ± 9 (*n* = 7)
Soleus	175 ± 16 (*n* = 10)	202 ± 14 (*n* = 9)	238 ± 22 (*n* = 11)	267 ± 37 (*n* = 10)
Force recovery (%)				
EDL	38 ± 3 (*n* = 8)	54 ± 6 (*n* = 9)	54 ± 3 (*n* = 11)	46 ± 3 (*n* = 6)
Soleus	50 ± 5 (*n* = 10)	68 ± 6 (*n* = 9)	61 ± 5 (*n* = 10)	52 ± 6 (*n* = 10)

*Note*: Maximum tetanic stress was normalized against muscle weight, fatigue resistance was determined as the time when half peak tetanus was reached, and force recovery as the percentage of force when compared with the maximum force produced during the fatigue protocol after 20 mins of rest. KW test showed no statistically significant difference among groups for all parameters (*p* > 0.01). Values are means ± SEM.

In control animals, exercise did not induced changes in the fatigue resistance of soleus muscles, suggesting that the endurance exercise training program was not intense enough to exert changes at this level (Table 3).

### 4.4 MyHC isoforms expression profile

In order to evaluate a first approach, the possible effect of the MetS on the fiber phenotype of EDL and soleus muscles, we determined the expression levels of the MyHC isoforms and the impact on fiber genetic composition.

**FIGURE 3:**
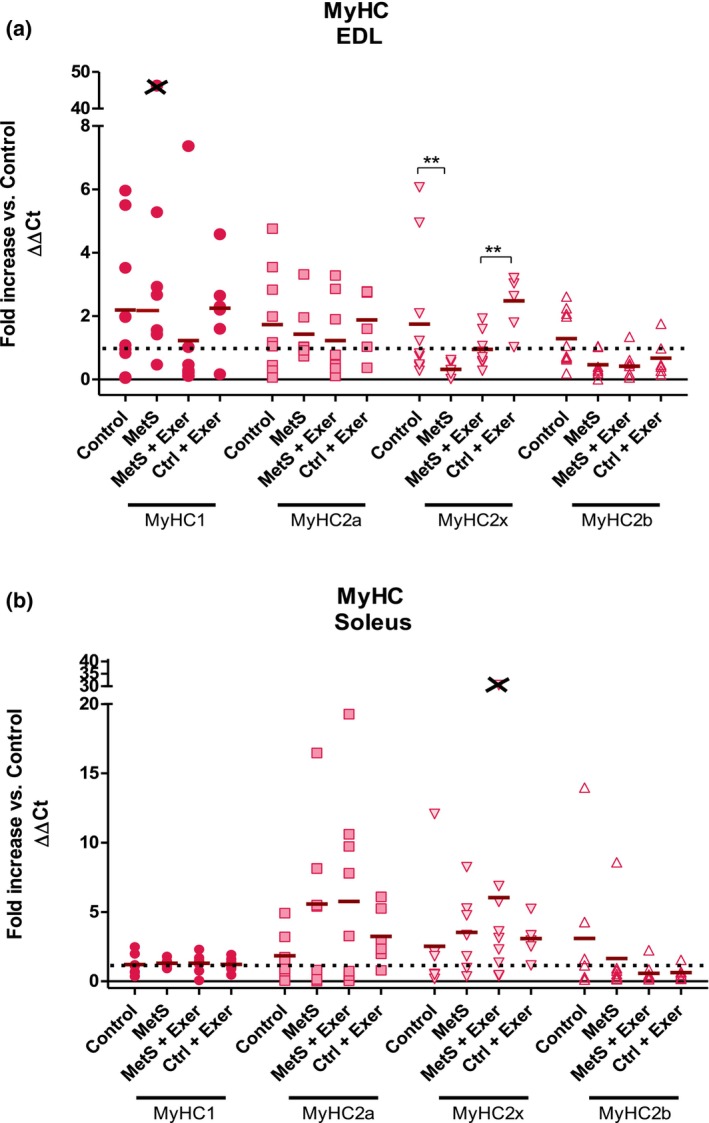
Muscle myosin heavy chain (MHC) gene expression. Fold increase when compared against versus control for MHC for EDL (a) or soleus (b). The percentage of each gene from the total is represented in (a) for EDL, and (b) for soleus. All the expression levels where normalized against the expression of the 18S gene. KW test showed statistically significant difference only on the MyHC2x for the EDL muscle (*p* < 0.01). Paired comparisons were made with MW test. ** = *p* < 0.01. Values are means ± SE. *n* = 6–10.

Figure 3 shows the relative expression levels of the MyHC isoforms from a 2^−ΔΔCt^ analysis. KW test showed no differences in all MyHC isoforms of the Soleus muscle in a 2^−ΔΔCt^ analysis (Figure 3b). KW test only showed statistically significant differences at isoforms 2x and 2b for the EDL muscles (Figure 3a). When evaluating MyHC expression, we found that in the EDL muscle, MetS induced a decreased expression of the fast isoform 2x (Figure 3a), and, thus a potential overall shift towards a slower phenotype, although protein expression was not determined. Endurance exercise was able to partially revert this phenotype change by increasing the expression of the fast‐intermediate 2x in the MetS EDL (Figure 3a). In order to strengthen these results further research should evaluate these changes at the protein level.

### 4.5 Expression of genes involved in the regulation of slow muscle phenotype

**FIGURE 4:**
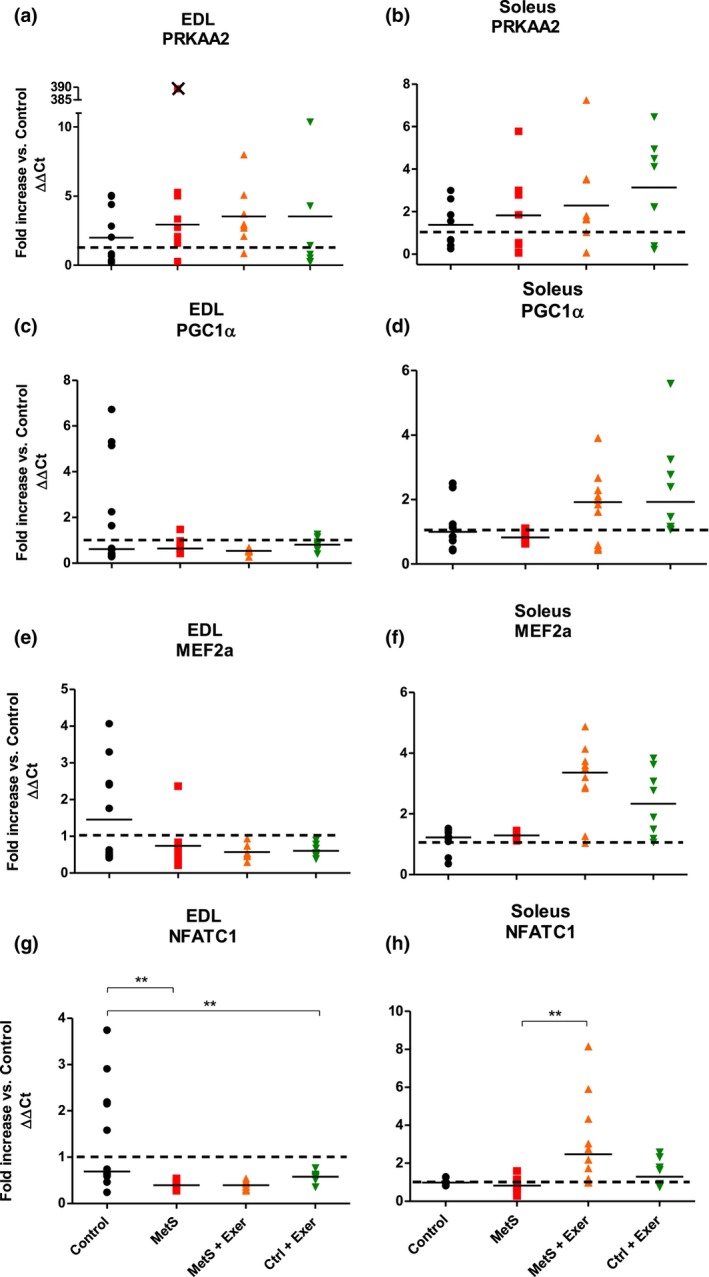
Gene expression levels of proteins involved in the regulation of the slow fiber phenotype. Fold increase when compared against the control for EDL (a, c, e, g) and soleus (b, d, f, h) muscles. KW test showed statistically significant difference among groups for Soleus and EDL on NFATC1 (*p* < 0.1). Paired comparisons were made with MW test. ** = *p* < 0.01. Medians are represented by the bar. *n* = 6–12. Crossed symbols on the graphs represent outliers and were not considered for the analysis.

To understand the mechanisms that mediate the shift of phenotype fiber in SM induced by MetS, we looked at the genes known to control fiber phenotypes. The KW analysis only showed statistically significant differences for the *NFATC1* expression on both EDL and soleus muscles (Figure 4). Suggesting that neither MetS nor endurance exercise had any effect over the expression levels of *PRKAA2*, *PGC1a* or *MEF2a* (Figure 4a–f). In the EDL muscle, *NFATC1* expression was reduced by MetS and this was not reverted in response to endurance exercise, while in the control animals, exercise training reduced its expression levels (Figure 4g).

On the soleus muscle, *NFATC1* expression was not modified by the MetS but, interestingly it was increased by exercise only on the MetS condition (Figure 4h).

These results suggest that the decrease in the fast MyHC2x isoform seen in the EDL muscle (Figure 2a) may be possibly regulated by other molecules, as no changes in the genes that upregulate the slow isoforms were seen.

### 4.6 Expression of genes involved in the regulation of fast muscle phenotype

**FIGURE 5:**
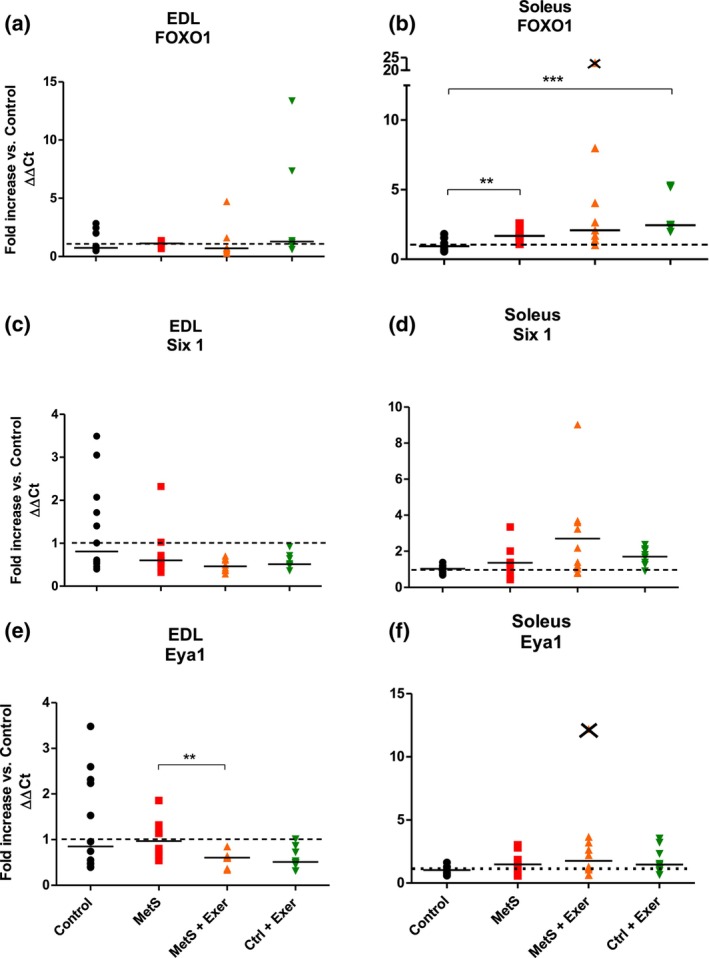
Gene expression levels of proteins involved in the regulation of the fast fiber phenotype. Fold increase when compared against the control for EDL (a, c, e) and soleus (b, d, f) muscles. KW test showed statistically significant difference among groups for Soleus FOXO1 (*p* < 0.01). Paired comparisons were made with MW test. ** = *p* < 0.01. Medians represented by the bar. *n* = 6–12. Crossed symbols on the graphs represent outliers and were not considered for the analysis.

Figure 5 shows the expression levels of genes involved in regulating the fast phenotype. The KW analysis showed statistically significant differences in *FOXO1* expression for the soleus muscle where MetS increased its expression as well as endurance exercise did in control animals (Figure 5b). *Six1* and *Eya1* remained unchanged in this muscle in all conditions (Figure 5d,f). MetS did not affect the expression of *FOXO1* nor *SIX1* in the EDL muscle, but interestingly, the expression of *EYA1* was significantly reduced in the MetS exercise group when compared with the MetS one (Figure 5a,c,e).

These results suggest that the potential shift into a slower phenotype of the EDL muscle due the MetS may not be a consequence of changes in the expression of these genes. Interestingly, *FOXO1*, that is thought to mediate the fast phenotype, seems to increase in the soleus muscle by effect of the MetS (when compared to control muscles) without modifying the MyHC profile (Figure 5b,d); therefore, this evidence indicates that their role on defining the SM phenotype and the response to exercise needs further research.

### 4.7 Activity of glycolytic and oxidative enzymes

Energy metabolic changes are associated to the development of MetS, key glycolytic and oxidative enzyme activities were evaluated in the muscles of the animals suffering MetS, as well as their response to the training protocol.

**FIGURE 6:**
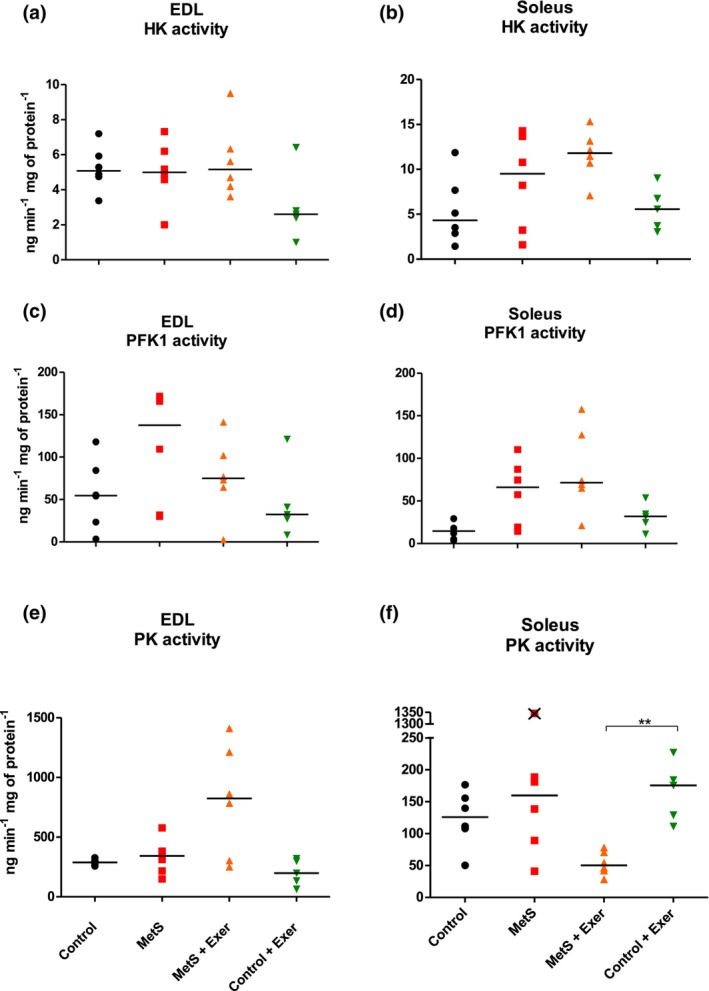
Activity levels of glycolytic enzymes. EDL (a, c, e) and soleus (b, d, f) muscles. HK‐2, hexokinase 2; PFK, phosphofructokinase‐1; PK, pyruvate kinase. KW test showed statistically significant difference among groups for soleus PK (*p* < 0.01). Paired comparisons were made with MW test. ** = *p* < 0.01. Medians are represented by the bar. *n* = 5–6. Crossed symbols on the graphs represent outliers and were not considered for the analysis.

Figure 6 depicts the activities of key enzymes of the glycolytic pathway. The KW analysis showed no statistically significant differences for HK and PFK1 for the soleus muscle (Figure 6b,d), while it did on the PK activity on the soleus of the control exercised animals, when compared to the MetS exercised animals this enzyme activity was increased (Figure 6f). No changes were seen on any of these enzymes on the EDL muscle (Figure 6a,c,e).

**FIGURE 7:**
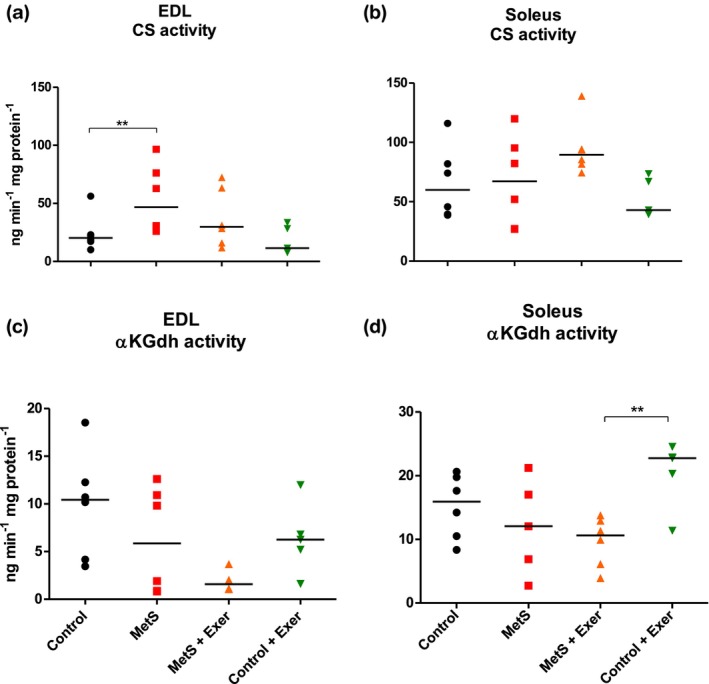
Activity levels of mitochondrial oxidative enzymes. For EDL (a, c) and soleus (b, d) muscles. CS, citrate Synthase; αKGDH, α‐ketoglutarate dehydrogenase. KW test showed statistically significant difference among groups for Soleus αKGDH (*p* < 0.01). For EDL CS a *p* = 0.056. Paired comparisons were made with MW test. ** = *p* < 0.01. Medians represented by the bar. *n* = 4–6.

Figure 7 shows the activities of key mitochondrial oxidative enzymes. The KW analysis showed statistically significant differences for αKGDH in the soleus muscle and a near significance (*p* = 0.056) for CS in the EDL. The activity of CS was increased as an effect of the MetS on the EDL muscle and this was only partially reverted as an effect of endurance exercise. Interestingly, the activity of αKGDH was not modified by the MetS or exercise at any condition for this muscle (Figure 7a,c). For the soleus muscle, the activity of the CS enzyme was not affected by the MetS or endurance exercise, suggesting that soleus adapted to the training and that exercise intensity should be increased to induce greater metabolical benefits (Figure 7b). As for the αKGDH on the soleus, we found that MetS had no effect on the enzyme activity levels, but interestingly the Control + exercise group did increase the activity of this enzyme (Figure 7d).

## 5 DISCUSSION

### 5.1 MetS makes the animals partially resistant to the metabolic and physical benefits of endurance exercise

Exercise is known to offer benefits to individuals who do not have metabolic diseases and these benefits on metabolic parameters are widely known.

MetS made the animals resistant to the beneficial effects of exercise, as it was only able to partially prevent the increase of the TG levels and glucose clearance after 3 h of administration and also, showed no significant changes on the AUC of the glucose tolerance test, indicating that IR is still present. Endurance exercise was able to successfully prevent weight gain and adiposity in sucrose fed animals (Table 2). Moderate intensity exercise seems to mildly improve metabolic and physical capacities of the SM as citrate synthase activity levels and VO_2 max_ of the control animals did not increase as previously reported (Siu et al., 2003), even when our animals ran at a constant speed of 20 m/min and at an inclination of 10°. It is important to note that the results obtained suggest that higher intensities and/or duration of exercise may be necessary to impact the alterations induced by MetS as previously shown (Groussard et al., 2019). Higher intensities of exercise may have exerted greater benefits as previously shown (Groussard et al., 2019), but the intensity of exercise performed by the animals in this study was sustained over 60% of maximum aerobic capacity (data not shown) similar to that recommended for obese persons (Organization WH, 2022). Consistently, this training protocol did not induced changes on the RQ of the MetS animals, implying that a transition from carbohydrate to lipid utilization during exercise may not be present. Also, it is important to note that the animals with MetS were not able to increase their maximum aerobic capacity to the levels of the control animals, but were able to perform better during the performance test. These results suggest that the better performance of these animals could be related to changes in the SM metabolic capacity rather than the cardiovascular function, which correlates better with the VO_2 max_. Other studies were also able to find an increased performance of animals during the first stages of MetS, that reduces significantly with aging (Liu et al., 2018) suggesting that, at early stages of the disease, SM adaptations to the altered state induce better physical performance and later, as the metabolic alterations progress, SM function starts to decline and consequently, also physical performance. The muscles of these animals may be changing their biochemical and mechanical properties and could be considered as a transition phenotype, in which oxidative metabolism and expression of proteins related with physical performance are prevented to adapt to the metabolic condition, however, in order to confirm this interpretation changes in the protein content of the muscles should be further evaluated.

Even though the animals and their muscles were resistant to some of the effects of exercise, moderate intensity exercise is strongly recommended to revert the effects of MetS, especially if coupled with healthier diets and lower caloric intake. Higher exercise intensities can be achieved after weight reduction and consequently, greater benefits can be obtained.

### 5.2 The fast muscle shift into a slower phenotype is partially reverted by endurance exercise

Although we only reported trends for changes in the muscle mechanical performance, we can expect that MetS could be modifying the contractile properties of the EDL muscle. It has been suggested that increase in maximum force could be related to specific forces applied to limb muscle, due to mass redistribution that comes from increased abdominal adiposity and causes changes in maximum force of different fibers of the muscles (Tallis et al., 2018; Warmington et al., 2000). This change has been observed in some studies (Eshima et al., 2017) but not others (Tallis et al., 2017) and may be dependent on the duration and/or the type of diet used to induce the MetS (Rodríguez‐Correa et al., 2020). Also, different molecular mechanisms may be implied in the phenomena.

In this study we determined transcriptional changes of several genes related with fiber composition transition, even when transcriptional changes not always relate to protein changes (particularly during exercise adaptation) (Miller et al., 2016), we think that these results may reflect a potential transition of fiber type composition, as smaller changes in transcription may produce pronounced changes at the protein level (Kirby, 2019) but it would be important to study changes at this level in the future. The possible shift of the EDL muscle into a slower phenotype could be a response to the altered metabolic properties that the MetS induces, in which mitochondrial activity is impaired and the cellular glucose uptake is reduced due to IR (Lee et al., 2006). Thus, this muscle may be increasing the number of mitochondria as a response to their reduced capacity for oxidative phosphorylation, and this may indirectly promote some changes in MyHC expression reducing the expression of the type 2b (Pereyra et al., 2022). These changes may also explain why trends suggest that EDL muscles with MetS responded to endurance exercise in a similar way as the soleus, by increasing the fatigue resistance, but it fails to explain why there was a tendency to increase the maximum tetanic stress. Changes in physical performance does not seem to be related to significant changes in *PGC1α* expression, and could possibly be explained by posttranscriptional regulation of this factor; phosphorylation of *PGC1α* plays an important role on its translocation to the nucleus and deacetylation on its function (Wright et al., 2007). Other molecules discussed below might be involved in this mitochondrial alteration, as well.

It is unclear if endurance exercise could prevent the shift into the slower phenotype of the EDL muscle, as shown by the changes in the expression of the MyHC type 2x; although it is important to note that, probably, during MetS, the changes in MyHC isoforms expression may have occurred from a shift from 2x to 2b types. But these changes do not seem to be related to the oxidative metabolism, and neither the expression levels of *AMPK* α2 subunit, *SIX1*, *EYA*1, nor the oxidative enzymes. However, AMPK phosphorylation, which controls the enzyme activity has been found altered during MetS (Ruderman et al., 2010); the results of this study do not rule out that changes that result from the phosphorylation state of the enzyme are involved.

In both exercise groups, we found no changes on the expression of *EYA1* suggesting it may not be related with the shift of fiber phenotype induced by exercise. Endurance exercise is known to mainly recruit slow muscles and this was the reason why we were expecting to find significant changes on the slow muscles, this could maybe be related to the exercise intensity used in this study. Our results suggest that fast muscles are altered at a higher degree than slow ones in this MetS model, and therefore we suggest that endurance exercise in combination with high intensity trainings may be more beneficial when preventing or treating MetS (Jin et al., 2018), as resistance exercise has been proven to improve fast muscles metabolism and help prevent metabolic alterations as well (LeBrasseur et al., 2011). In fact, a positive correlation between fast type 2b fibers expression and obesity has been found (Kriketos et al., 1997). It is also important to note that the fiber composition greatly differs between humans and rodents, since humans present a higher proportion of slow fibers than rats, so the effect of MetS and exercise on the fast muscles should be further investigated (Armstrong & Phelps, 1984; Bloemberg & Quadrilatero, 2012). We suggest that these muscles may be in a transition state mediated by the MetS, and that both endurance and resistance training, may partially prevent such transition.

### 5.3 MetS dissociates the metabolic response to exercise from the phenotype fiber shifts in soleus

When evaluating the expression of the MyHC isoforms, we were unable to find the shift into the faster phenotype which has been reported (Tallis et al., 2017). Although there is evidence that this shift does not always occur, the changes in contractile and fiber type profiles induced by obesity have been supported in a review (Tallis et al., 2018). It is possible that these changes depend on several factors such as diet duration and type, and that it affects different muscles in various manners. Even when we did not observe a shift into a faster phenotype, we found an increase expression of *FOXO1* due to MetS, which has been found to induce the fast phenotype of the muscles (Pudi et al., 2007), and could be one of the factors playing a role on the alterations suffered by slow muscles during the MetS, as reported in other studies (Eshima et al., 2017; Simi et al., 1991; Tallis et al., 2017). Chronic endurance exercise has been found to decrease *FOXO1* levels in fast and slow muscles, suggesting a regulation towards the slower phenotype (Yuan et al., 2011). Interestingly, our results showed the opposite, that is, an increase of the transcription levels of this factor in the slow muscles due to MetS and exercise, in both the experimental and control groups. The role of *FOXO1* on SMs during the MetS should be further investigated as it could be playing a key role in the SM fiber type transition.

Exercise was not able to promote the expression of *PGC1α*, *NFATC1* and *MEF2a*, and therefore muscles from MetS animals were not able to benefit from the metabolic remodeling mediated by these genes, as *PGC1α* may be failing to respond to exercise in the mitochondria. It appears that the metabolic response of the muscle to MetS is altered, since it seems that the number of mitochondria remains unchanged, as reflected by CS, while the rest of oxidative metabolism does not change in response to exercise. This is especially true for *PGC1α*, which is the main manager of mitochondrial biogenesis.

The expected changes on oxidative capacity due to endurance exercise were not seen, which are related to the function of *MEF2a* and *PRKAA2*, thought to promote mitochondrial biogenesis (Carvajal et al., 2003; Sousa et al., 2018). Changes in the metabolic capacity of the muscles were not evident, suggesting that no metabolic adaptations to MetS during exercise occur, highlighting the importance of not assuming phenotype changes on the muscles only by evaluating one of these properties, and that assessing multiple aspects is important when evaluating fiber type transition.

It is important to note that posttranscriptional regulation, and interactions with other molecules such as those related to Ca^2+^ handling and fatty acid metabolism, may be involved in the response of MetS muscles to exercise (Carvajal et al., 2014; Guadalupe‐Grau et al., 2018). This model resembles early stages of the MetS; therefore, we think that fast muscles suffer greater alterations at early stages; to cope with the diminished glucose uptake induced by IR. As the alterations grow in severity, slow muscles start being unable to adapt at the altered oxidative metabolism and suffer greater alterations, as seen in other rodent models, such as those in which MetS is induced by genetic mutation or high‐fat diets (Rodríguez‐Correa et al., 2020). All this may partially explain inconsistencies found in literature, particularly within the shifts into opposite phenotypes of SMs (reviewed in 37).

## 6 CONCLUSIONS

Our results highlight the importance of studying the affections of the fast muscles during the MetS, and also the effects that different types of exercise exert on it. It is important to note that the muscles of the body are rarely composed by one single, but rather by a mixture of fiber types; therefore, it is important to assess the effect that changes on the fast fibers may exert on whole‐body metabolism during MetS and exercise, as it may be more important than previously thought. Also, here we highlight that MetS and endurance exercise affect metabolic, mechanical and structural properties of the muscles, and should all them be evaluated when determining alterations in this tissue.

### 6.1 Limitations

We understand that histological techniques have been the golden standard to determine fiber type shift in muscle, however, in order to these changes to be evident, an initial shift in gene expression is headed. Indeed, our results does not show a radical shift in the type of fiber, that is, a slow to fast shift, however gene expression changes may determine changes in the properties and function of the muscle in the MetS animals, as revealed by our results. These changes are present along with metabolic changes, potentially resulting in a “transition” type of fiber that represent the response to the MetS condition in which metabolic and/or structural composition of the fiber may vary in different ways. Nonetheless, histological and protein changes may reveal if gene expression impact the morphological composition of the muscle and deserve further attention. It is also important to note that multivariate statistical analysis increases the astringency of the tests and could therefore mask the biological effects of these conditions, as shown by some trends in our results.

## AUTHOR CONTRIBUTIONS

ERC conducted experiments, analyzed and discussed results, wrote the draft manuscript and elaborated figures. KC conceived and designed the project and experiments, supervised execution of experiments, analyzed and discussed results. ERC and KC reviewed and approved the manuscript.

## CONFLICT OF INTEREST STATEMENT

Authors declare no conflict of interest.

## Data Availability

Data will be made available on request.
